# Artificial intelligence for the detection of acute myeloid leukemia from microscopic blood images; a systematic review and meta-analysis

**DOI:** 10.3389/fdata.2024.1402926

**Published:** 2025-01-17

**Authors:** Feras Al-Obeidat, Wael Hafez, Asrar Rashid, Mahir Khalil Jallo, Munier Gador, Ivan Cherrez-Ojeda, Daniel Simancas-Racines

**Affiliations:** ^1^College of Technological Innovation, Zayed University, Abu Dhabi, United Arab Emirates; ^2^Internal Medicine Department, Medical Research and Clinical Studies Institute, The National Research Centre, Cairo, Egypt; ^3^NMC Royal Hospital, Abu Dhabi, United Arab Emirates; ^4^Department of Clinical Sciences, College of Medicine, Gulf Medical University, Ajman, United Arab Emirates; ^5^Department of Allergy and Immunology, Universidad Espiritu Santo, Samborondon, Ecuador; ^6^Respiralab Research Group, Guayaquil, Ecuador; ^7^Centro de Investigación de Salud Pública y Epidemiología Clínica (CISPEC), Universidad UTE, Quito, Ecuador

**Keywords:** artificial intelligence, acute myeloid leukemia, blood images, machine learning, neural networks, meta-analysis

## Abstract

**Background:**

Leukemia is the 11^th^ most prevalent type of cancer worldwide, with acute myeloid leukemia (AML) being the most frequent malignant blood malignancy in adults. Microscopic blood tests are the most common methods for identifying leukemia subtypes. An automated optical image-processing system using artificial intelligence (AI) has recently been applied to facilitate clinical decision-making.

**Aim:**

To evaluate the performance of all AI-based approaches for the detection and diagnosis of acute myeloid leukemia (AML).

**Methods:**

Medical databases including PubMed, Web of Science, and Scopus were searched until December 2023. We used the “metafor” and “metagen” libraries in R to analyze the different models used in the studies. Accuracy and sensitivity were the primary outcome measures.

**Results:**

Ten studies were included in our review and meta-analysis, conducted between 2016 and 2023. Most deep-learning models have been utilized, including convolutional neural networks (CNNs). The common- and random-effects models had accuracies of 1.0000 [0.9999; 1.0001] and 0.9557 [0.9312, and 0.9802], respectively. The common and random effects models had high sensitivity values of 1.0000 and 0.8581, respectively, indicating that the machine learning models in this study can accurately detect true-positive leukemia cases. Studies have shown substantial variations in accuracy and sensitivity, as shown by the Q values and I^2^ statistics.

**Conclusion:**

Our systematic review and meta-analysis found an overall high accuracy and sensitivity of AI models in correctly identifying true-positive AML cases. Future research should focus on unifying reporting methods and performance assessment metrics of AI-based diagnostics.

**Systematic review registration:**

https://www.crd.york.ac.uk/prospero/#recordDetails, CRD42024501980.

## 1 Introduction

Leukemia is a form of blood cancer that has several unique features. It is the 11th most prevalent type of cancer worldwide, accounting for approximately 2.5% and 3.1% of all new cancer incidences and mortality in 2020, respectively (Bray et al., 2018; Sung et al., 2021). Acute leukemia can be classified into two types: myeloid and lymphoid. Acute lymphocytic leukemia (ALL) is the most prevalent leukemia in children, whereas acute myeloid leukemia (AML) is the most common malignant blood malignancy in adults (Okikiolu et al., 2021). Hematologists use numerous laboratory techniques to detect and diagnose leukemia. The diagnostic methods begin with a microscopic morphological inspection of the peripheral blood smear (PBS) and bone marrow (BM) slides, followed by immunophenotyping and cytogenetic analysis to further confirm the diagnosis of leukemia (Hegde et al., 2018; Bain, 2005). Other methods include molecular cytogenetics, long-distance inverse polymerase chain reaction (LDI-PCR), and Array-based Comparative Genomic Hybridization (aCGH). However, owing to the time and cost requirements of these complicated techniques, microscopic blood tests are the most common method for identifying leukemia subtypes (Ahmed et al., 2019).

Traditional blood disorder detection based on visual inspection of blood smears under a microscope is time-consuming, error-prone, and restricted by the hematologist's physical acuity (Amin et al., 2015). Therefore, an automated optical image processing system is necessary to facilitate clinical decision-making. Medical image analysis has gained popularity in the biomedical world owing to its potential to enhance disease detection, diagnosis, and decision-making accuracy (Ben-Suliman and Krzyżak, 2018; Elsayed et al., 2023; Chaurasia et al., 2024; Li et al., 2023). Several medical image-based and machine-learning algorithms have been proposed to identify leukemia, reduce the need for human intervention, and ensure accurate clinical diagnosis (Hegde et al., 2019; Baig et al., 2022; Bibi et al., 2020; Karar et al., 2022).

Artificial Intelligence (AI) is a broad term for devices that imitate human intellect. Machine learning (ML), a subset of AI, refers to teaching computer algorithms to generate predictions based on experience (Hunter et al., 2022). It includes *k*-nearest neighbors (KNN), support vector machine (SVM), random forest, Extreme Gradient Boosting (XGBoost), and artificial neural network (ANN) (Yue et al., 2022). Deep learning (DL) is a subset of ML in which complex architectures similar to the linked neurons of the human brain are created (Hunter et al., 2022). Deep neural networks (DNNs), autoencoder networks (AEs), generative adversarial networks (GANs), recurrent neural networks (RNNs), and convolutional neural networks (CNNs) are examples of deep learning methodologies (Patterson and Gibson, 2017). CNN is among the most widely used deep learning (DL) networks. The key advantage of CNN over its predecessors is that it automatically recognizes significant traits without human intervention, making them the most widely used (Alzubaidi et al., 2021). CNN-based computerized deep learning algorithms have demonstrated outstanding performance in the detection, segmentation, and classification processes involved in medical imaging (Nasr-Esfahani et al., 2016). These include multiple predefined architectures with varying degrees of complexity, such as AlexNet (Krizhevsky et al., 2017), EfficientNet (Tan and Le, 2019), InceptionNet (Szegedy et al., 2015), ResNet (He et al., 2016), and DenseNet (Huang et al., 2017).

Our systematic review and meta-analysis aimed to analyze and cover all AI-based approaches for the detection and diagnosis of AML. We reviewed multiple recent studies, including DL techniques, intending to identify the overall accuracy and sensitivity of these methods using microscopic PBS images.

## 2 Methods

This systematic review and meta-analysis was conducted according to The Preferred Reporting Items for Systematic Reviews and Meta-Analyses (PRISMA) statement guidelines and all steps were performed with strict adherence to the Cochrane Handbook of Systematic Reviews and Meta-analysis. It was registered with PROSPERO under registration number CRD42024501980.

### 2.1 Search strategy

We conducted a thorough search using relevant keywords, such as “acute myeloid leukemia,” “artificial intelligence,” “deep learning,” “machine learning, ” and other related terms. The medical databases searched included PubMed, Web of Science, and Scopus from inception until December 2023. No timeframe or language restrictions were applied.

The detailed search strategy can be found in Supplementary material 1.

### 2.2 Study selection

Screening was conducted by two independent authors in two steps: Title/Abstract screening, followed by full-text screening. Any conflicts were resolved through consensus or group discussion.

Our inclusion criteria were as follows: (1) utilization of human AML peripheral blood smear samples, (2) employment of AI techniques for diagnosing/classifying AML, (3) reporting of performance metrics, recall (sensitivity), and accuracy, which served as our main outcome measures; and (4) separate metrics were provided for AML diagnosis, not an overall model accuracy.

Studies that did not meet these criteria were excluded to ensure a focused and relevant analysis. The exclusion criteria were as follows: (1) studies that discussed irrelevant topics or diagnostic methods, such as acute promyelocytic leukemia (APL), myelodysplastic syndrome (MDS), flow cytometry, protein detection, or microarray gene algorithms; (2) studies investigating the accuracy of image segmentation into blasts or leukocyte images rather than whole images for disease classification; (3) studies with the outcome of disease prognosis or identifying disease subtypes (M1, M2, etc.); and (4) studies with incomplete data, case reports, review articles, editorials, conference/meeting abstracts, guidelines, and letters.

### 2.3 Methodological quality assessment

The degree of bias was assessed using Quality Assessment of Diagnostic Studies 2 (QUADAS-2). Comprehensively, we identified four domains: patient selection, index test, reference standard, flow, and timing. The first three domains were assessed for applicability. The risk of bias was judged to be “low,” “high,” or “unclear.” Signaling questions were included to help reach a judgment regarding the risk of bias.

### 2.4 Data extraction

Data were extracted independently by two authors using Microsoft Excel. Any disagreements were resolved by consensus between the authors. The following data were extracted for each study: number of patients/samples, total number of images used in the validation sets after augmentation, classification task (binary or multiclass), databases used with their reference standards, use of classifiers, application of transfer learning, and type of validation used.

In addition, the name of each author, publication year, country where the study was conducted, type of study (prospective or retrospective), and the design and algorithm architecture names of AI systems were also retrieved.

## 3 Strategy for data synthesis and statistical analysis

For the meta-analysis, we used the “metafor” and “metagen” libraries in R to analyze the accuracy of the different models used in the studies. The dataset for this analysis consisted of 24 models across 10 studies, each employing a variety of classifiers including CNNs and SVMs. We used both common- and random-effects models for data analysis and forest plots to improve data visualization. The random-effects model allowed for the testing of variability in effect sizes between the studies. The Z-value was used to determine the statistical significance of the findings along with the *p*-value. A larger z-value (in absolute terms) corresponds to a smaller *p*-value, indicating that the observed effect is less likely to occur by chance. The threshold for statistical significance was set at *P* < 0.05.

To assess heterogeneity, the I^2^ statistic was calculated to quantify the percentage of total variation across studies; values above 60% indicated high heterogeneity. The H^2^ statistic, an estimate of the ratio of total variability to sampling variability, was additionally quantified alongside the “Q-value” which measures the degree of variability in the results of different studies where a high H^∧^2 value (>1.5) and large Q-value with a low *p*-value (*p* < 0.05) suggests the presence of significant heterogeneity. The Restricted Maximum Likelihood (REML) method was used to further evaluate the estimated amount of total heterogeneity (tau^∧^2). The standard error (SE) and the square root of Tau^∧^2 (tau) were used to quantify the uncertainty or variability in the estimate of the heterogeneity, where a smaller SE and tau indicate more precise estimates. Heterogeneity was considered statistically significant when the two-tailed *p*-value was < 0.05.

To evaluate the performance of the AI models, we conducted a meta-analysis of studies that provided sufficient information on accuracy and sensitivity. If a study provided several tables or values for the different algorithms used, each model was treated as an independent variable.

Funnel plots were generated and visually inspected to check for publication bias.

## 4 Results

### 4.1 Study selection

**Figure 1:**
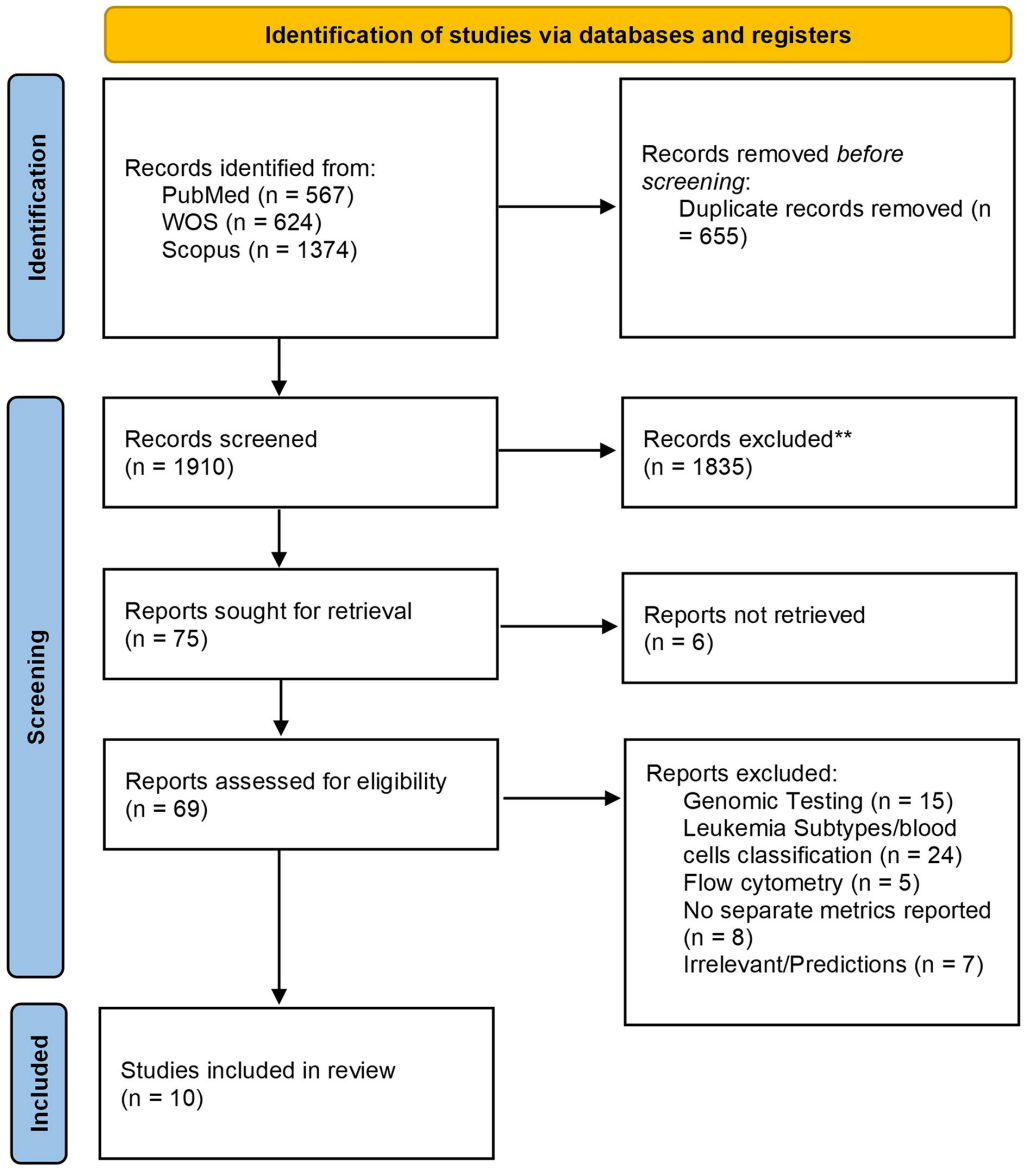
The Preferred reporting items for systematic reviews and meta-analyses (PRISMA) 2020 flow chart depicting the screening process for included studies.

A total of 2,565 records were recovered, 655 of which were removed as duplicates. Following title and abstract screening, only 75 articles were deemed acceptable for full-text screening. Finally, 10 studies were eligible and included in our systematic review and meta-analysis. A detailed PRISMA diagram illustrating the study selection steps and the full PRISMA checklist are presented in Figure 1 and Supplementary material 2, respectively.

### 4.2 Baseline characteristics of included studies

We evaluated 10 studies (Baig et al., 2022; Bibi et al., 2020; Karar et al., 2022; Sakthiraj, 2022; Shalini and Viji, 2023; Veeraiah et al., 2023; Shawly and Alsheikhy, 2022; Kazemi et al., 2016; Nagiub et al., 2020; Abhishek et al., 2023) On AML detection that were performed between 2016 and 2023. These studies have been conducted in various countries including Pakistan, Saudi Arabia, the United States, India, Iran, and Egypt. They employed both binary and multiclass classification tasks to distinguish between different types of leukemia and healthy samples. Two of these studies (Kazemi et al., 2016; Nagiub et al., 2020) used a heterogeneous image set, including both PBS and bone marrow data; however, they met all the necessary inclusion criteria to participate in our analysis.

Regarding the type of AI algorithm used, most studies have depended on DL algorithms. Specifically, CNNs were used in seven studies (Baig et al., 2022; Bibi et al., 2020; Sakthiraj, 2022; Shalini and Viji, 2023; Shawly and Alsheikhy, 2022; Nagiub et al., 2020; Abhishek et al., 2023), GANs in two (Karar et al., 2022; Veeraiah et al., 2023), and SVM in one (Kazemi et al., 2016). For the selection of datasets, five studies (Bibi et al., 2020; Karar et al., 2022; Sakthiraj, 2022; Shalini and Viji, 2023; Nagiub et al., 2020) depended on images from online datasets such as the American Society of Hematology Image Bank (ASH-bank) and the Acute Lymphoblastic Leukemia Image Database for Image Processing **(**ALL-IDB). At the same time, the rest of the studies either used local data images from hospitals, and laboratories, or another online dataset (namely, The Kaggle site) (Shawly and Alsheikhy, 2022).

The classification was mostly multi-class classification to stratify images into AML, ALL, normal, or other leukemia types, while only three studies performed binary classification (Shawly and Alsheikhy, 2022; Kazemi et al., 2016; Nagiub et al., 2020). Transfer learning was utilized in four studies, and classifiers in five studies. Detailed characteristics of the included studies, including the study design, chosen dataset, number of images used (while applying image augmentation or not), and name of the AI algorithm tested, among others, can be found in Tables 1, 2.

**Table 1:** Characteristics of the included studies.

**References**	**Country**	**Classification task (binary or multi-class)**	**Databases/datasets**	**Design**	**Reference standard of database**	**AML samples/patient count**	**Number after data augmentation/image selection**
Baig et al. (2022)	Pakistan	Multi (AML vs. ALL vs. MM)	Leukemia Diagnostics at Munich University Hospital, ISBI 2019 public dataset and MiMM_SBILab Dataset.	Retrospective (hospital data and online dataset)	Expert-based and diagnoses per standard guidelines	118 patients	307 AML random images
Bibi et al. (2020)	Pakistan, Saudi Arabia	Multi (AML vs. ALL vs. MM)	ASH image bank and ALL-IDB	Retrospective (online dataset)	Peer-reviewed images by hematologists and expert oncologists	55 samples	1,194 AML
Karar et al. (2022)	Saudi Arabia	Multi (AML vs. ALL vs. Healthy)	ASH image bank and ALL-IDB	Retrospective (online dataset)	Peer-reviewed images by hematologists and expert oncologists	77 AML	No augmentation
Sakthiraj (2022)	USA	Multi (AML vs. ALL vs. CML vs. CLL vs. Healthy)	ASH image bank	Retrospective (online dataset)	Peer-reviewed images by hematologists	56 AML samples	1,198 AML
Shalini and Viji (2023)	India	Multi (AML vs. ALL vs. CML vs. CLL vs. Healthy)	ASH image bank and ALL-IDB	Retrospective (online dataset)	Peer-reviewed images by hematologists and expert oncologists	104 blood smear images	No augmentation
Veeraiah et al. (2023)	India, Saudi Arabia	Multi (AML vs. ALL vs. CML vs. CLL)	N/A	N/A	N/A	301 AML random images	No augmentation
Shawly and Alsheikhy (2022)	Saudi Arabia	Binary (AML vs. ALL)	The Kaggle site	Retrospective (online dataset)	Expert oncologists	1,016 AML images (for validation)	No augmentation
Kazemi et al. (2016)	Iran	Binary (AML vs. normal)	Shariati Hospital pathology laboratories (9 males and 8 females, aged 16–69 years old)	Prospective	Clinical, blood, and bone marrow examination.	27 peripheral blood smear and bone marrow slides of AML patients	165 AML images
Nagiub et al. (2020)	Egypt	Binary (AML vs. normal)	AML-IDB from the Hematology Unit, Department of Clinical Pathology, Assiut University Hospitals, Egypt (from 2017 to 2019)	Prospective (hospital data)	Clinical data (clinical history and examination) and laboratory data (morphological, cytochemical, and immunophenotyping assessment) according to hematologist's experience	206 AML images	No augmentation
Abhishek et al. (2023)	India	Multi (AML vs. ALL vs. CML vs. CLL vs. Healthy)	The Hematology section, the Department of Pathology, AIIMS Patna	Prospective (between May 2019 and February 2022)	Two experts' opinions consensus	19 patients	250 AML images

ALL, Acute Lymphoblastic Leukemia; AML, Acute Myelogenous Leukemia; MM, Multiple Myeloma; CML, Chronic myeloid leukemia; CLL, Chronic Lymphocytic Leukemia; N/A, Not available; ASH-bank, The American Society of Hematology (ASH) Image Bank; ALL-IDB, Acute Lymphoblastic Leukemia Image Database for Image Processing; AML-IDB, AML Image Database; AIIMS, All India Institute of Medical Sciences.

**Table 2:** Types of models used and their specifications.

**References**	**AI Model (SVM, CNN, etc.)**	**Algorithm architecture name**	**Transfer learning**	**Classifier**	**Validation**	**Dataset**	**Main outcomes and conclusion**
Baig et al. (2022)	Hybrid CNN models	CNN-1, CNN-2	Yes	5 types: SVM, Bagging ensemble, total boosts, RUSBoost, and fine KNN	N/A	-Acute lymphoblastic leukemia: 293 sample -Acute myeloid leukemia:307 sample -Multiple Myeloma:301 sample	This research developed an automated diagnosis tool for ALL, AML, and MM. The dataset was pre-processed where they found that the output images had already been segmented. Two CCN models were trained in parallel to extract features. The CCA Fused approach is used to concatenate these derived features. The classifier receives fused vectors (SVM, Bagging ensemble, total boost, RUSBoost, Fine KNN). Using the Bagging ensemble design, it achieved a 97.04 percent accuracy. As a result, pathologists may find that this procedure aids in effective diagnosis.
Bibi et al. (2020)	CNN models (ResNet-34 and DenseNet-121)	ResNet-34 and DenseNet-121	Yes	Not needed	IV	-ALL: Before augmentation: 181 After augmentation: 1,079 - AML Before augmentation: 55 After augmentation: 1,194 - CLL Before augmentation: 38 After augmentation: 840 - CML Before augmentation: 57 After augmentation: 1,243	In the proposed framework, an IoT-enabled microscope uploads the blood smear images to the leukemia cloud. Leukemia is diagnosed by using the ResNet-34 or DenseNet-121 models. It is observed that the diagnosing power of ResNet-34 and DenseNet-121 supersedes all the previous approaches. By using data augmentation techniques, ResNet-34 and DenseNet-121 both process large numbers of image patterns. After diagnosis, the result is sent to the doctor's computer where s/he provides medical care based on the test report through the IoMT framework. Furthermore, the proposed framework facilitates the patients in pandemics such as COVID-19.
Karar et al. (2022)	Auxiliary classifier with Generative Adversarial Network model (AC-GAN)	AC-GAN	No	Not needed	Cross-validation	-ALL: 179 -AML: 77 -Normal: 189	The proposed IoMT framework utilizes cloud computing services to provide accurate online leukemia tests, saving hematological efforts and lowering the required computing resources. An advanced deep learning architecture, the AC-GAN model, was developed to identify leukemia and its two sub-classes. Compared with previous works, the semi-supervised AC-GAN model showed promising classification results for acute leukemias.
Sakthiraj (2022)	Hybrid Convolutional Neural Network with Interactive Autodidactic School (HCNN-IAS) algorithm	HCNN-IASO	No	Softmax-CNN layer classifier (based on ResNet-34 and DenseNet-121)	IV	-Before augmentation: Healthy: 190 CML: 58 CLL: 30 AML: 56 ALL: 182 -After augmentation: Healthy: 1,291 CML: 1,244 CLL: 845 AML: 1,198 ALL: 1,082	The proposed approach is used to generate results and to accurately identify and detect them. The data augmentation technique involved is utilized to practice big datasets and thus it processes large Leukemia images. The features from Leukemia datasets are extracted by using our proposed HCNN and further the attention layer in the HCNN is exploited to fuse the extracted features. The softmax layer of HCNN acts as a classifier and therefore it classifies the leukemia dataset into several subtypes. Furthermore, the accuracy of classification is optimized by utilizing Interactive autodidactic school optimization techniques. Finally, the optimized outcomes are sent to the medical institution/hospital via an IoMT platform for further processing. Based on the results retrieved, the physician/doctor provides a diagnosis to the patients.
Shalini and Viji (2023)	Hybrid Squeeze-and-Excitation Networks (SENet)-based CNN	SENet-CNN	No	Not needed	IV	The dataset included ALL, AML, CALL, CML, and healthy. Specifications of each category weren't mentioned.	The SENet-CNN models are used to determine the leukemia diagnosis. Images of stained blood smears were segmented into WBC nuclei, and then the article extracted pertinent features to identify leukemia. The proposed SENet-CNN method's accuracy of 99.98% is more than that of the existing classification methods.
Veeraiah et al. (2023)	Mayfly optimization with Generative Adversarial Network (MayGAN)	MayGAN	No	Not needed	IV	-Acute lymphoblastic leukemia (ALL): 294 -Acute myeloid leukemia (AML): 301 -Chronic lymphocytic leukemia (CLL): 304 -Chronic myelogenous leukemia (CML):301	This research created an automatic diagnosis tool for four classes. Utilizing the suggested methods, the dataset was pre-processed to reduce noise and blurriness and improve image quality. This work discovered that the output photos had already been segmented during pre-processing. The strategy is valid and avoids the need for image segmentation. It is found that the proposed MayGAN achieves 99.8% of accuracy, 98.5% of precision, 99.7% of recall, 97.4% of F1-score, and 98.5% of Dice Similarity coefficient (DSC).
Shawly and Alsheikhy (2022)	8-layer CNN	AlexNet	No	SVM	IV	10,500 blood samples: 70% of the dataset for training purposes, 15% testing, and 15% validation. The dataset included ALL, AML, and healthy.	The proposed method can detect and classify ALL and AML cancer with high precision and accuracy as proved by the conducted experiments. Hence, it can be used in hospitals and healthcare centers to support and assist hematologists and laboratory technicians in their tasks. In addition, the developed algorithm reaches an accuracy of nearly 99% in detection and classification.
Kazemi et al. (2016)	SVM	Binary and multi-SVM	No	Not needed	k-fold cross-validation	-ALL: 750 -AML: 750	The proposed methods are relatively simple yet this algorithm demonstrates satisfactory performance for the diagnosis between AML patients and normal persons and also for the detection of prevalent subtypes of AML. Hence, the proposed algorithm can be used as an assistant diagnostic tool for pathologists.
Nagiub et al. (2020)	Pre-trained CNN models	Alexnet, VGG16, GoogleNet, ResNet101, and Inception-v3.	Yes	Not needed	IV	−206 images of patients with leukemia -206 images of healthy normal controls.	The statistical measures of the Inception-v3 performance revealed promising results. The sensitivity, specificity, and accuracy of Inception-v3 reached 99.98% for detection and classification performed between the two classes in the data set: normal control and leukemia. Inception-v3 required only 0.2273 s to test each image in AML-IDB. Thus, Inception-v3 is recommended as a robust automated method for leukemia detection. It can act as a second opinion in the disease diagnosis after a manual evaluation of the disease by a hematologist to increase the consistency of the laboratory practice on the daily diagnostic routine.
Abhishek et al. (2023)	Pre-trained CNN models	MobileNet, DenseNet121, ResNet152V2, VGG16, Xception, and InceptionV3	Yes	SVM, RF, and FCL	5-fold cross-validation	The database had 1,250 images. There are 250 images of each class (normal, CLL, ALL, CML, and AML.) Microscopic images of two sub-types of ALL (T-ALL and B-ALL) and images of six sub-types of AML (M0, M1, M2, M3, M4, and M5) were included	Pre-trained VGG16 along with SVM helped in achieving an accuracy of 81%. When LTCL of VGG16 is also fine-tuned, it helps in better classification of acute Leukemia along with chronic ones. Hence, the overall classification accuracy of classifiers also improved on the combined dataset. FCL as a classifier achieved an accuracy of 80% whereas SVM as a classifier achieved an accuracy of 84%. The features responsible for classifying an image to a particular class are visualized with the help of class-specific heatmaps generated by the Grad-CAM technique.

CNN, Convolutional Neural Network; SVM, Support Vector Machine; KNN, K-nearest Neighbor; CCA, Canonical Correlation Analysis (CCA); IV, Internal Validation; ALL, Acute Lymphoblastic Leukemia; AML, Acute myelogenous leukemia; CML, Chronic Myelogenous Leukemia; CLL, Chronic Lymphocytic Leukemia; MM, Multiple Myeloma; IoT, Internet of Things; IoMT, Internet of Medical Things; AML-IDB, AML Image Database; RF, Random Forest; FCL, Fully Connected Layers; LTCL, Last Three Convolutional Layers; N/A, Not available.

Table 3 summarizes the definitions, advantages, and limitations of different AI models included in our study.

**Table 3:** Advantages and limitations of different AI models.

**Model**	**Definition**	**Advantages**	**Limitations**	**References**
CNN-	The convolutional neural network is a machine learning model effective in image recognition	-Accuracy -Efficiency and Automation	-Need large training datasets -Need powerful computational approaches -High computational cost -Generalizability issues	Salehi et al., 2023; Sayyad et al., 2023
Bagging ensemble model	Bagging uses multiple models for prediction. Each model is trained on a subset of the data set, and the predictions are averaged or combined	-Reduce overfitting -Stable and generalizable model	-Poor sensitivity to outliers -High computational cost -High bias	Mohammed and Kora, 2023; Ziyadullaev et al., 2024
Total boost model	Boosting is a method for creating an accurate classifier from simpler classifiers	-Accuracy -Generalizability	-Overfitting -Power consumption	Ohn-Bar and Trivedi, 2016; Chaudhary et al., 2024
KNN	K Nearest Neighbors (KNN)models are memory-based models that are used for regression and classification tasks	-Simplicity -Efficient for large datasets -Flexible	-Computational Complexity -Sensitive to noisy data	Farid et al., 2022; Acito, 2023
SVM	Support Vector Machine is a machine-learning model used in classification	-Few model parameters -Simplicity and flexibility in classification -Effective for non-linear classification	-High computational cost -Kernel selection problems -Performance issues in the unbalanced datasets	Cervantes et al., 2020; Bhavsar and Panchal, 2012; Shammi et al., 2023; Diana et al., 2023
LPBoost	Linear programming boosting algorithm combines weak classifiers through linear programming to obtain a linear combination	-Good at classification	-Computational cost -Time consuming	Zhang et al., 2021; Liu and Vemuri, 2011
RUSBoost	RUSBoost algorithm combines random under-sampling and boosting techniques	-Simplicity -Short model training time -Suitable for imbalanced data	-Data loss during model development	Tanha et al., 2020; Seiffert et al., 2008
DenseNet	DenseNet is a deep-learning algorithm consisting of multiple layers	-Easy to train -Improved gradients and information flow -Reduced overfitting	-Slow training time -High computational cost	Zhou et al., 2022; Wang et al., 2020; Huang et al., 2022
ResNet	Residual Neural Network (ResNet) is an artificial neural network used for image recognition	-Accuracy -Ability to extract and categorize critical elements from images	-Prone to overfitting -Degradation problems	Duta et al., 2021; Ebrahimi and Abadi, 2021
AC-GAN	Auxiliary Classifier Generative Adversarial Network is a generative network that is used for various learning problems	-Simplicity -Accuracy -Suitable for imbalanced data sets -Generates high-quality images	- Low intra-class diversity -Interpretability problems -High computational cost	Mudavathu et al., 2018; Gomathi et al., 2024; Hou et al., 2022
HCNN-lASO	-The Least Absolute Shrinkage and Selection Operator (LASSO) is a machine-learning tool -Hierarchical CNN is an effective model for knowledge transfer using its hierarchical structure	-Solve multicollinearity problems -Enhanced interpretation -Feature extraction -Suitable for large-scale images -Robustness	-Computational complexity -Problems with feature selection	Kim et al., 2019; Xi et al., 2023 Khalajzadeh et al., 2013; Zhao et al., 2024
SENet	SENet is a convolutional neural network structure that uses Squeeze-and-Excitation Networks to increase interconnections of channels and function	-Reduced overfitting -Enhanced model performance -Flexibility	-Computational cost	Pragy et al., 2019; Liu et al., 2018; Hu et al., 2018
GAN	Generative Adversarial Networks (GANs) are generative AI models that generate and classify data	-Suitable for complicated data -Enhance categorization	-Model training consumes time -Gradient vanishing -Instability issues	Sharma et al., 2024
AlexNet	AlexNet is a CNN-based approach used for image categorization	-High efficiency -High speed -Robustness	-High computational cost	Amanollah et al., 2023
VGG	Visual Geometry Group is a multiple-layer deep neural network architecture	-Simplicity -Feature learning ability	-Limited scalability -Larger parameters need large memory -High computational cost	Guan et al., 2019; Zhou et al., 2021; Zakaria and Mohmad Hassim, 2023
GoogleNet	- GoogLeNet is a deep learning structure that combines and extracts features from the input	-Extract rich image features -High accuracy	-High computational performance -Model's implementation consumes time	Chen et al., 2023
Inception	Inception is a convolutional architecture that extracts features from images	-Suitable for complex structures -Accuracy	-Complex -Difficult to modify -Underfitting	McNeely-White et al., 2020; Jing et al., 2023
RF	A random forest is a supervised machine learning that consists of several trees	-Suitable for large datasets -High accuracy -Flexibility -Fast to train	-Overfitting -Not suitable for unbalanced data -Sensitive to hyperparameters	Zhu, 2020

CNN, Convolutional Neural Network; KNN, K-Nearest Neighbors; SVM, Support Vector Machine; LPboost, Linear Programming Boosting; RUSBoost, Random Under-sampling Boost; ResNet, Residual Network; AC-GAN, Auxiliary Classifier Generative Adversarial Network; HCNN, Hierarchical Convolutional Neural Network; LASSO, Least Absolute Shrinkage and Selection Operator; GAN, Generative Adversarial Network; VGG, Visual Geometry Group; RF, Random Forest.

### 4.3 Assessment of the potential for bias (Quality)

**Figure 2:**
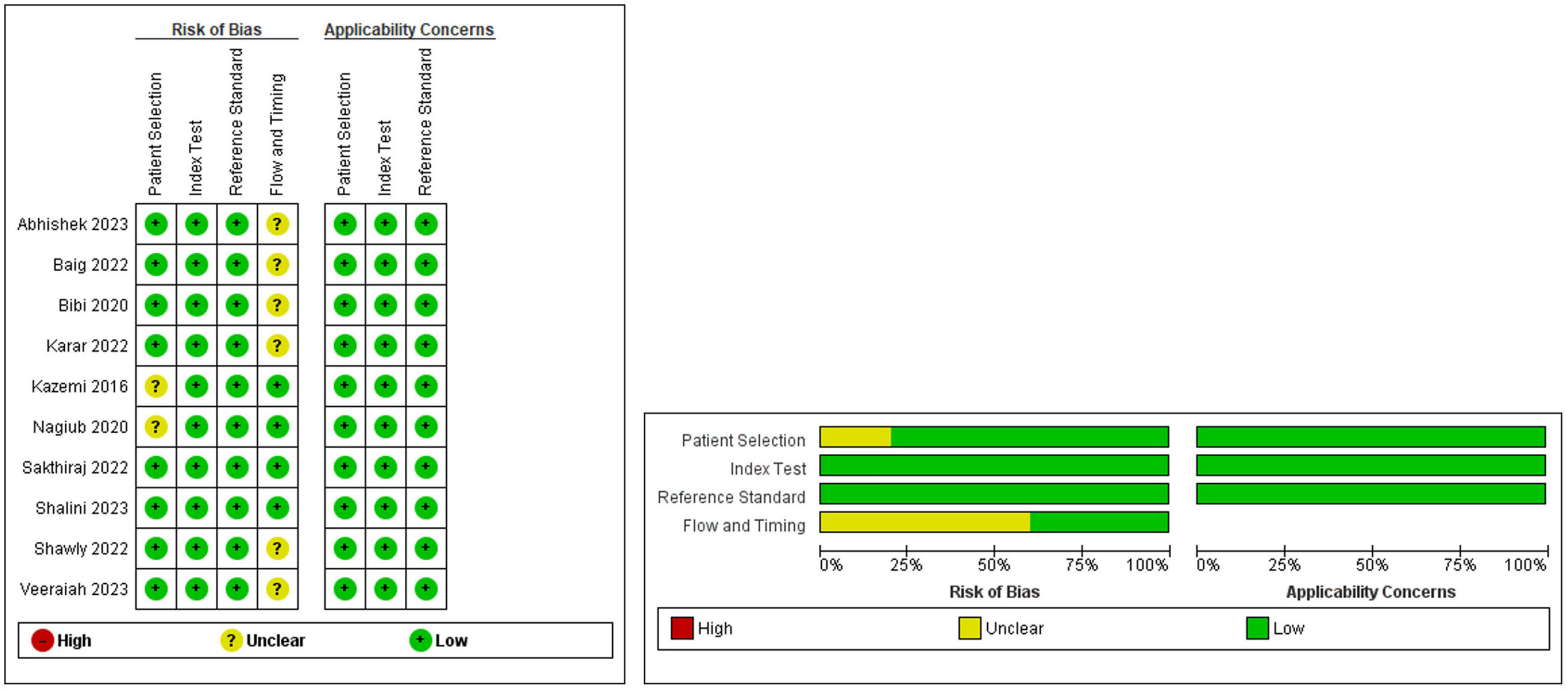
Quality Assessment of included studies using QUADAS-2 tool.

Quality assessment using the QUADAS-2 tool revealed an overall low risk of bias and a low risk of applicability concerns, with some unclarity regarding the flow and timing domains (Figure 2).

### 4.4 Data synthesis and meta-analysis

#### 4.4.1 Accuracy

**Figure 3:**
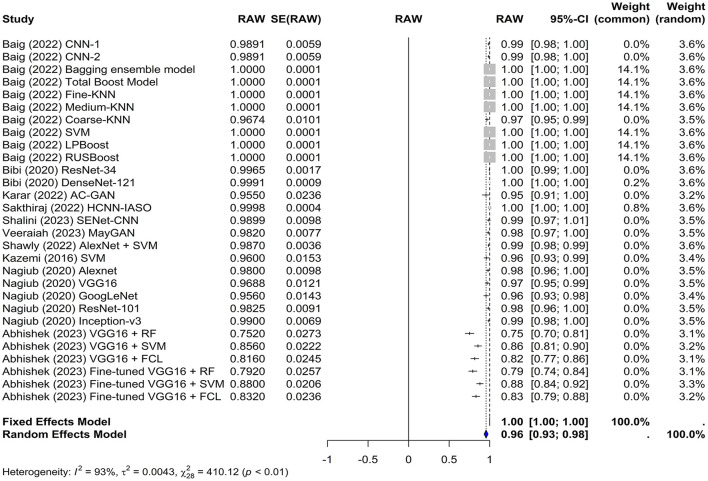
Forest plot for analyzing the accuracy of the different models used across the studies. CI, confidence interval.

The common effect model yielded an accuracy of 1.0000 [0.9999; 1.0001], whereas the random-effects model yielded an accuracy of 0.9557 [0.9312; 0.9802]. In the random-effects model, the estimate of the overall accuracy was 0.9557 with a standard error of 0.0125. The z-value was 76.5840, and the *p*-value was < 0.0001, indicating that the overall accuracy was significantly different from chance (Figure 3).

The test for heterogeneity resulted in a Q-value of 410.1247 with 28 degrees of freedom, indicating significant heterogeneity among the studies (*p* < 0.0001). The I^2^ and H^2^ statistics were 100.00% and 94,583.49, respectively, suggesting a high level of heterogeneity. Furthermore, heterogeneity among studies was quantified using tau^∧^2 and tau. The Tau∧2 value was 0.0043 with a standard error (SE) of 0.0012, and the tau (square root of the estimated Tau^∧^2 value) was 0.0659.

These results demonstrate the potential of artificial intelligence in detecting leukemia with high accuracy. However, the high level of heterogeneity suggests that the accuracy may vary depending on the specific characteristics of the study, such as the type of classifier used and whether transfer learning was employed.

#### 4.4.2 Sensitivity

**Figure 4:**
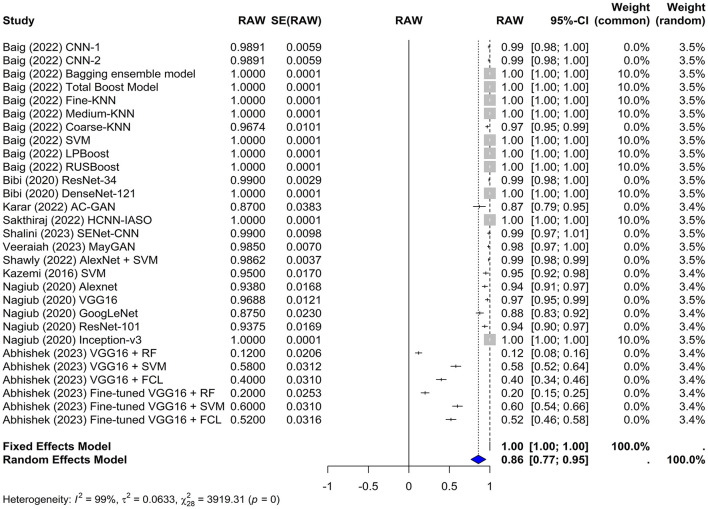
Forest plot for analyzing the sensitivity of the different models used across the studies. CI, confidence interval.

In this meta-analysis, both the common and random effects models yielded high sensitivity values of 1.0000 and 0.8581, respectively, suggesting that the machine learning models used in the studies were effective in correctly identifying true positive cases of leukemia. In the random-effects model, the overall sensitivity was estimated to be 0.8581 with a z-value of 18.33 and a *p*-value of < 0.0001, which indicates that this sensitivity significantly differs from chance (Figure 4). Several models achieved 100% sensitivity in the diagnosis of leukemia such as KNN, LPboost, Inception, and DenseNet-based models. The VGG16+RF and the fine-tuned VGG16+RF models in Abhishek et al. (2023) had the lowest sensitivity (12% and 20%, respectively).

The test for heterogeneity yielded a Q-value of 3,919.31 with 28 degrees of freedom. A *p-*value of 0 indicates significant heterogeneity among the studies, suggesting that the variability in study outcomes is due to real differences in effect sizes rather than chance. The I^2^ statistic was 99.3%, indicating a high level of heterogeneity, which was further confirmed by an H^2^ value of 11.83.

Furthermore, the Tau^∧^2 was 0.0633, with an SE of 0.0012 and tau of 0.2516, which provided additional information about the heterogeneity among the studies.

### 4.5 Publication bias

**Figure 5:**
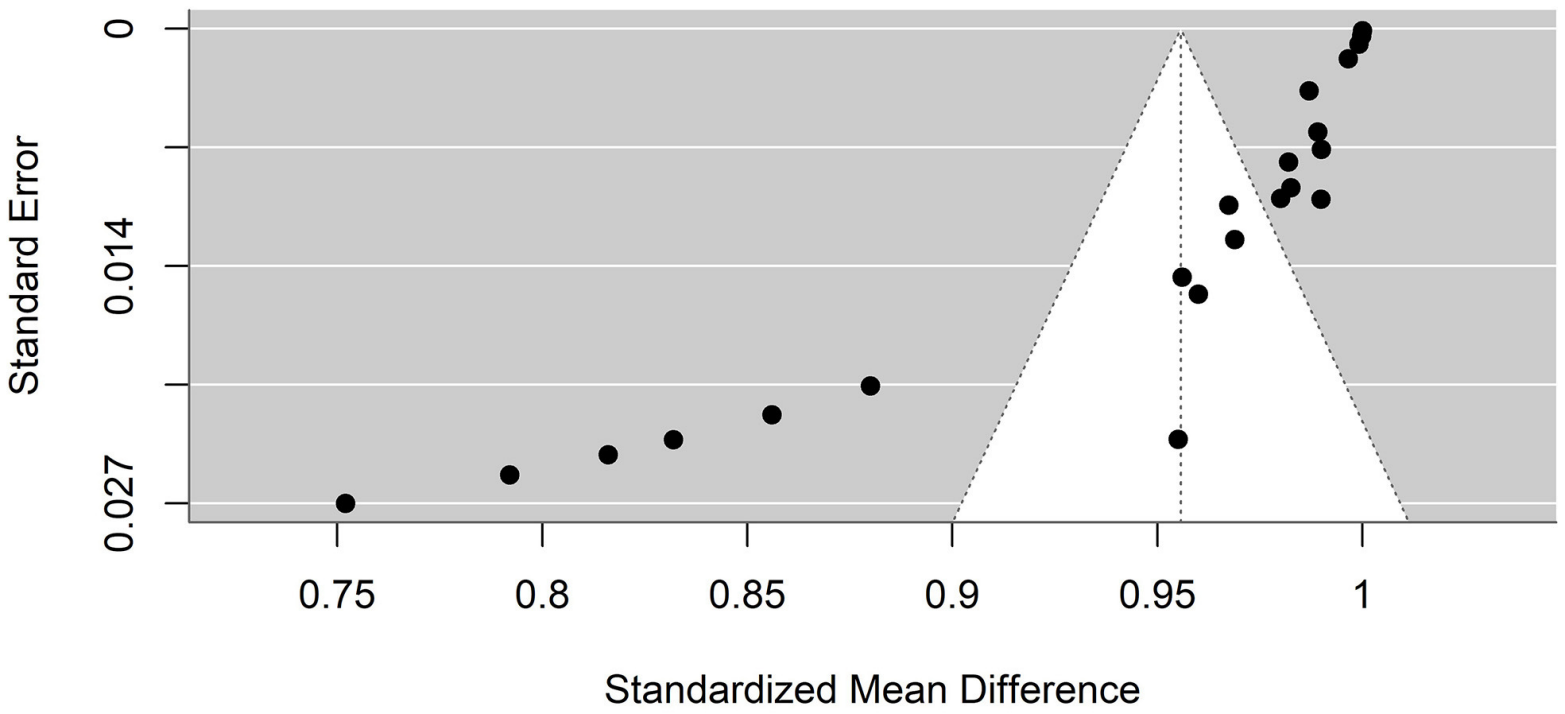
Precision funnel plot of the estimated effects from studies on artificial intelligence model performance accuracy.

**Figure 6:**
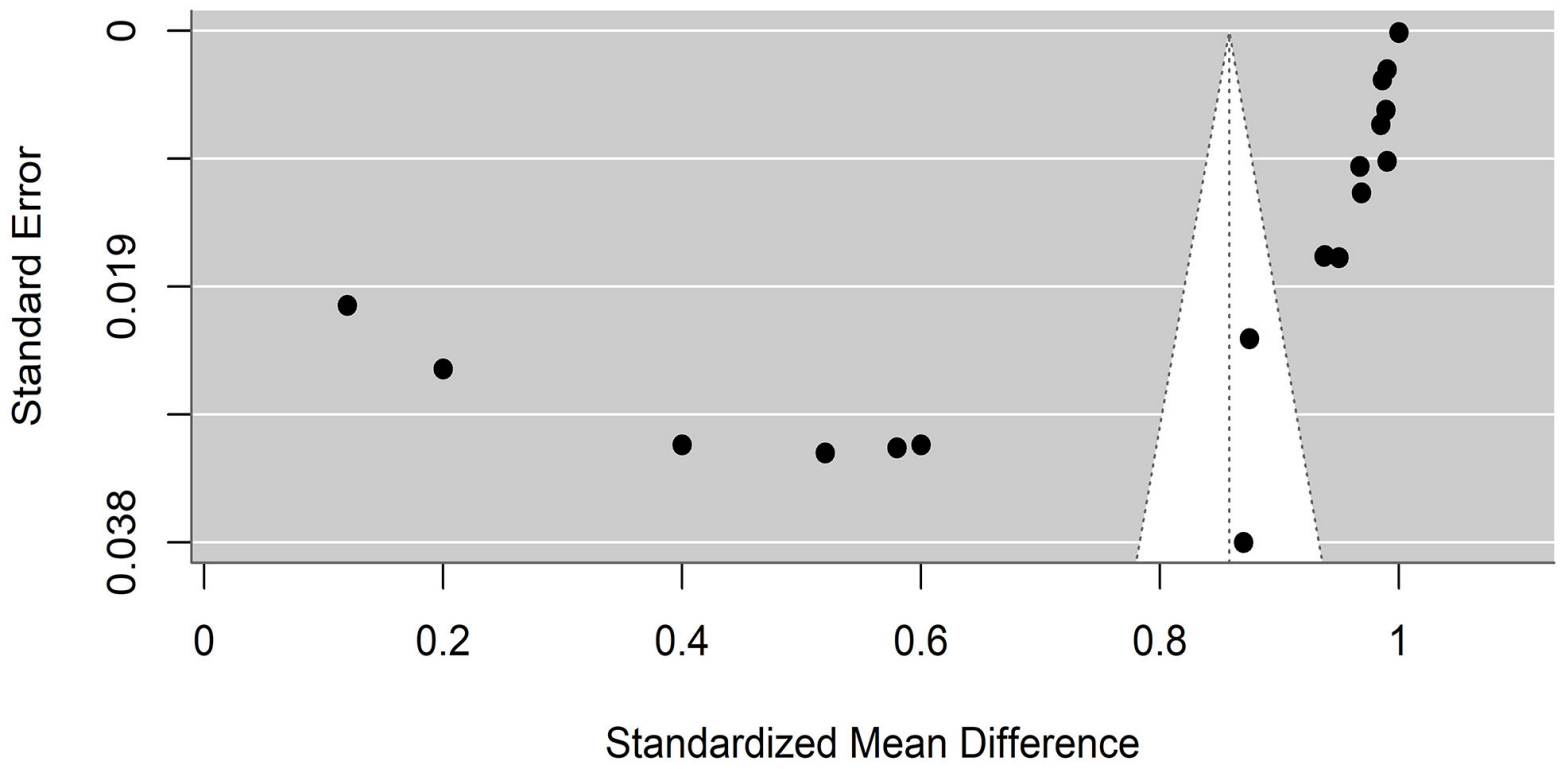
Precision funnel plot of the estimated effects from studies on artificial intelligence model performance sensitivity.

Funnel plots were created to detect potential biases or systematic heterogeneity. The asymmetry observed in the plots suggests potential publication or other bias, indicating that smaller studies with positive outcomes are more likely to be published. Several studies appeared outside the funnel shape. This may be due to a small sample size, poor study design, or heterogeneity (Figures 5, 6).

## 5 Discussion

Our meta-analysis aimed to analyze the diagnostic accuracy of AI methods in identifying and diagnosing AML, which revealed significant findings regarding the performance of machine-learning models in such detection. Both the common effects and random effects models demonstrated high accuracy, with values of 1.0000 and 0.9557 respectively. However, there was significant heterogeneity among the studies, as indicated by a Q-value of 410.1247 and I^2^ statistic of 100%. Additionally, both models showed high sensitivity for correctly identifying true-positive cases of leukemia, with values of 1.0000 and 0.8581, respectively. Nevertheless, sensitivity also demonstrated significant heterogeneity among the studies, as shown by a Q-value of 3,919.31, and an I^2^ statistic of 99.3%.

The significant heterogeneity in the accuracy results suggests that the accuracy of each model may vary depending on the specific characteristics of each study, such as the type of classifier used and whether transfer learning is employed. Baig et al. (2022) initially tested two CNN models for proper identification of AML from ALL or healthy cells. Subsequently, they applied multiple classifier models using fusion methods, such as the Bagging Ensemble and the RUSboost, aiming to combine the complex feature vectors of CCN-1 and CNN-2, thus improving the prediction performance. On the other hand, other studies, such as Bibi et al. (2020), Kazemi et al. (2016), and (Nagiub et al., 2020) only focused on the main ML model used without any further classifications, where they yielded satisfactory results. Such mixed approaches have resulted in varying ranges of accuracy and subsequent overall heterogeneity.

Remarkably, Baig et al. (2022) used traditional ML models. This was justified by the need to minimize the computation of the network used. Training a deep learning network can take several hours or even days, whereas traditional machine learning models require a few minutes. The use of a DL model such as a CNN while training it using a traditional ML classifier displayed quite remarkable results compared to DL. This can be attributed to the limited dataset sizes, where training complex DL models usually requires larger datasets (Sarker, 2021). Furthermore, leukemia microscopic images can be complex, containing nuanced morphological and textural characteristics that may be difficult for DL models to extract reliably. Such factors could potentially contribute to traditional ML methods, which sometimes outperform DL methods.

Transfer Learning was another common variable among the included studies. Some authors prefer to work with pre-trained models to speed up the results and generate faster outcomes. In particular, one model is that of Abhishek et al. (2023), who tested multiple pre-trained CNN models and subsequently chose the top-performing model (VGG16) for further fine-tuned analysis. However, other studies preferred to train their models from scratch, including Shalini and Viji (2023) who trained a squeeze-and-excitation network (SENet)-based CNN model on a hybrid dataset of blood smear images by combining both the ASH-bank and the ALL-IDB to complement the data. Heterogeneity is further magnified through these vast differences between testing models; however, this is expected due to the continuous evolution of the ML and DL worlds. Notably, most studies demonstrated closely related statistics, except for the models used by Abhishek et al. (2023), which demonstrated lower values for both accuracy and sensitivity. However, this most likely cannot be attributed to transfer learning as a concept in general, as various other studies have used it, and the results are promising. A possible rationale for the poor performance of these models could be the variation in the training dataset domain between the CNN models and the deep transfer learning dataset. Their study involved deep transfer learning using a microscopic blood smear dataset; therefore, there is a potential for negative transfer because the pre-trained CNN models were trained on the ImageNet dataset, which only comprises real-life images, resulting in the overall low accuracy of the models.

A few important elements that can have a significant impact on the AI model performance are feature extraction, data augmentation, data source and size, and model design. For instance, traditional machine learning techniques frequently depend on domain-specific feature engineering, in which experts manually identify and extract pertinent features from data (Gibert et al., 2022). On the other hand, deep learning models can automatically learn features by utilizing the hierarchical structure of the network; nevertheless, the model architecture and training data affect the quality of the learned features (Gibert et al., 2022). Ideally, a combination of both approaches could significantly enhance detection systems, as previously mentioned by Baig et al. (2022). Finally, image augmentation was a common factor in almost half of the included studies (Baig et al., 2022; Bibi et al., 2020; Sakthiraj, 2022; Kazemi et al., 2016; Abhishek et al., 2023) and performed better in training their sets on a larger number of samples. This helped to increase the diversity and size of the training dataset, which is an important aspect for DL models to yield better results. Additionally, the origin of the data, whether from one or more sources, can also have an impact on how well the model handles variances and real-world situations. Over half of the included studies utilized online datasets, which could have been beneficial in enhancing their sensitivity and accuracy, as they included data from multiple sources rather than a single area/hospital.

Internet of Medical Things (IoMT) is a common term observed in three studies included in our review (Bibi et al., 2020; Karar et al., 2022; Sakthiraj, 2022). It is essentially a medical device that communicates with Wi-Fi and smart computer networks (Ud Din et al., 2019). Smart medical gadgets use sensors and computational resources to provide healthcare in various settings, including homes, clinics, hospitals, healthcare facilities, and basic communities (Khan et al., 2020). Consequently, they are linked to cloud platforms for data analyses and processing. Linking patients to doctors and securely transferring medical data reduces the strain on health systems, allowing for the accurate remote examination, diagnosis, and treatment of many disorders (Awan et al., 2021; Almogren et al., 2021). Bibi et al. developed a model utilizing ResNet-34 and DenseNet-121, with promising accuracy (Bibi et al., 2020). Karar et al. (2022) established a GAN classifier integrated within an IoMT framework for multiclass classification of ALL, AML, and normal blood images. Finally, the last study (Sakthiraj, 2022) used a hybrid Convolutional Neural Network with an interactive autodidactic school (HCNN-IAS) algorithm, which has multi-performance effects in terms of feature extraction, fusing, and classification operations. The proposed methodology allowed for higher classification accuracy in terms of the detection of different leukemia classes, with an accuracy of approximately 99%. All these approaches utilizing the IoMT architecture allow doctors to provide medical care based on test results supplied to their computers after diagnosis, which in turn is of promising value for optimized patient care.

Different methods of outcome reporting are one noticeable concern that varied across the studies. For instance, some studies reported the area under the curve (AUC) and false positive rate, whereas others produced results in terms of precision and F-1 scores. Therefore, it is necessary to define precise reporting guidelines for diagnostic accuracy studies evaluating AI procedures to unify the reporting methods among similar studies and to aid in performing homogenous meta-analyses. Examples of anticipated work in progress include STARD-AI (Sounderajah et al., 2020) and TRIPOD-AI (Collins and Moons, 2019). The QUADAS-2 assessment tool was used to systematically assess the risk of bias and applicability in diagnostic accuracy studies. However, this tool was not specifically designed for DL diagnostic accuracy studies. The unique nature of ML and DL studies requires the creation of a novel specific and unified quality assessment tool for all healthcare-related AI tools (Aggarwal et al., 2021).

AI has been used for image diagnosis in similar studies in which comparable findings were found. For instance, Sampathila et al. (2022) tested a CNN model for diagnosing ALL, and the results showed a high performance, as evidenced by an accuracy of 95.54%, specificity of 95.81%, and sensitivity of 95.91%. Additionally, Ghaderzadeh et al. (2021) performed a systematic review of studies classifying leukemia using ML on PBS images and found an average accuracy of >97%. Furthermore, Rawat et al. (2017) introduced a computer-assisted classification framework using SVM, which achieved a maximum accuracy of 99.5% for screening AML and ALL blast cells. Deep convolutional networks are also used in detecting the ratio of WBCs in peripheral blood smear analysis. The proposed model relied on hyperspectral imaging technology (HSI), which combines conventional imaging and spectroscopy to produce 3-dimensional data. The model achieved 97.72% accuracy in the WBC classification (Wang et al., 2021). Wang et al. (2017) developed an AI-based model to identify lymphoblast and lymphocytes and diagnose ALL. The model combined spectral and spatial information achieving 92.9% accuracy (Wang et al., 2017). This highlights the potential of AI models in the diagnosis of different types of leukemia.

However, all of these studies focused on detecting either ALL alone or leukemia in general, with no prior meta-analyses evaluating the diagnostic accuracy of whole PBS images for AML. This highlights the uniqueness of our analysis in both the detection of AML and the use of whole images rather than leukocyte/blast-cell images.

## 6 Limitations and strengths

Our study has several limitations. First, a high level of heterogeneity was observed between the included studies. This is probably because of the continuous change in the ML and AI worlds, where multiple methods of data augmentation, classification, transfer learning, and feature extraction are used. The varying sample sizes and number of images used between studies are another limitation that could affect the results. Most of the included studies additionally utilized ASH-bank as the main dataset for model training; thus, the generalizability of our findings regarding diagnostic performance in different clinical settings is limited. Another drawback is that the counts needed to reconstruct the 2 × 2 tables of results for each study were not always provided; thus, analysis of more diagnostic metrics, such as specificity, was limited. Moreover, one of the main differences between these studies was the application of a data augmentation technique to the training and testing sets. Such an application can result in a misleadingly higher accuracy than the genuine value; therefore, the results are not always realistic. Finally, the potential publication bias was presented, where most of the models with positive results are likely to be the ones published disregarding others that might affect our interpretation of the overall AI accuracy.

On the other hand, to the best of our knowledge, this is the first systematic review with a meta-analysis specifically on the accuracy of AI models in diagnosing AML. Previous studies have frequently focused on single-cell classification or used preprocessed images, limiting applications to real-world situations. Our focus on the analysis of whole PBS images mitigated this issue and enhanced overall accuracy.

## 7 Conclusion and future directions

In conclusion, our systematic review and meta-analysis found an overall high accuracy and sensitivity of AI models in correctly identifying true-positive cases of Acute Myeloid Leukemia. This is the first study to compare artificial intelligence-related studies discussing the diagnosis of AML in particular rather than ALL or Leukemia diagnoses in general.

Future research should focus on assessing multiple performance measures to assess every possible outcome related to the tested model. The unification of accuracy, sensitivity, and specificity for each cancer type, rather than an overall average, would be more valuable in allowing for the proper critical appraisal of each model in terms of properly identifying AML.

Additionally, additional work related to the advancement of DL-based diagnostic tools as an IoMT approach is highly intriguing. Cancer treatment is a complicated process and the ability to diagnose samples through an accurate IoMT device with fewer hospital visits, especially during epidemics and pandemics like the recent COVID-19, would be extremely beneficial, especially if the future models delve deeper into the diagnosis of different subtypes.

## 8 Summary

Leukemia is the 10th most common type of cancer globally, and acute myeloid leukemia (AML) is the most common malignant blood cancer in adults.

Microscopic blood testing is the most common method used to identify leukemia subtypes. An automated optical image processing system employing artificial intelligence (AI) has recently been used to aid clinical decision-making, although its performance and accuracy remain unclear.

We aimed to assess the effectiveness of all AI-based techniques in the detection and diagnosis of AML using a systematic review and meta-analysis.

We discovered that AI models are often quite accurate and sensitive for properly recognizing true-positive cases of AML.

Future research should focus on harmonizing AI-based diagnostic reporting techniques with performance assessment criteria.

## Data Availability

The original contributions presented in the study are included in the article/Supplementary material, further inquiries can be directed to the corresponding author.
