# Supplementary material for: Artificial intelligence for the detection of acute myeloid leukemia from microscopic blood images; a systematic review and meta-analysis
Source: Front Big Data. 2025 Jan 17;7:1402926. doi: 10.3389/fdata.2024.1402926 (PMC11782132; doi:10.3389/fdata.2024.1402926)
Supplement: Supplementary file 2 [file Table_2.docx]

**Detailed Search Strategy for AI in AML diagnosis:**

("Artificial intelligence" OR "machine learning" OR "deep learning" OR "image analysis" OR "natural language processing" OR "Computational Intelligence" OR "Machine Intelligence" OR "Computer Reasoning" OR AI OR "Computer Vision Systems" OR "Computer Vision System" OR "AI-based" OR "Knowledge Acquisition" OR "Knowledge Representation" OR "Supervised machine learning" OR "Unsupervised Machine Learning" OR "Expert systems" OR "Fuzzy Logic" OR "Neural Networks" OR "Artificial Neural Networks" OR "Artificial Neural Network" OR " Medical Informatics" OR "Random Forest" OR "SVM" OR "Support vector machine") AND ("Acute myeloid leukemia" OR "acute myelocytic leukemia" OR "acute myelogenous leukemia" OR "acute granulocytic leukemia" OR "acute non-lymphocytic leukemia" OR AML OR ANLL OR "Acute Nonlymphocytic Leukemia" OR "Acute nonlymphoblastic leukemia" OR "Acute non-lymphoblastic leukemia" OR "Acute Myeloblastic Leukemia")
